# Black Soldier Fly Larva Fat in Broiler Chicken Diets Affects Breast Meat Quality

**DOI:** 10.3390/ani13071137

**Published:** 2023-03-23

**Authors:** Bartosz Kierończyk, Mateusz Rawski, Zuzanna Mikołajczak, Piotr Szymkowiak, Kinga Stuper-Szablewska, Damian Józefiak

**Affiliations:** 1 Department of Animal Nutrition, Faculty of Veterinary Medicine and Animal Science, Poznań University of Life Sciences, Wołyńska 33, 60-637 Poznań, Poland; 2 Laboratory of Inland Fisheries and Aquaculture, Department of Zoology, Faculty of Veterinary Medicine and Animal Science, Poznań University of Life Sciences, Wojska Polskiego 71c, 60-625 Poznań, Poland; 3 Department of Chemistry, Faculty of Wood Technology, Poznań University of Life Sciences, Wojska Polskiego 75, 60-625 Poznań, Poland

**Keywords:** poultry, dietary fat, *Hermetia illucens*, insect, breast muscle

## Abstract

**Simple Summary:**

Insect fat has been well established as a viable dietary energy source that can replace commonly used and environmentally harmful plant and animal fats in livestock nutrition. The use of black soldier fly (BSF) larvae fat in broiler chicken diets resulted in comparable growth performance parameters to those achieved with dietary soybean oil. Thus, there is a possibility to fully replace soybean oil without detrimental effects. This is mainly because the energy densities of the abovementioned dietary fats are similar. However, there is still a need to expand our knowledge about the impact of using *H. illucens* fat in poultry diets on the quality of the final products, including meat. The results of the present study confirmed that BSF larvae fat affects the fatty acid profile of breast muscle, particularly in terms of n3 deficiency. Simultaneously, no significant effect on atherogenic and thrombogenic indices was noticed. Moreover, insect fat does not impair the physicochemical indices, i.e., color, pH, and water-holding capacity, to such an extent as to limit the choice of the product by the consumer.

**Abstract:**

This study aimed to evaluate the dose-dependent effect of black soldier fly (BFL) larvae fat inclusion in broiler chicken diets on breast meat quality. Four hundred 1-day-old male birds (Ross 308) were assigned to the following four treatments (10 replicates with 10 birds each): HI0, a basal diet without dietary fat inclusion, and HI03, HI06, and HI09, basal diets enriched with 30 g/kg, 60 g/kg, and 90 g/kg of BSF larvae fat, respectively. Principal component analysis showed noticeable differentiation between the selected plant, animal, and insect-origin dietary fats. The BSF fat exhibits a strong relationship with saturated fatty acids (SFAs), resulting in a high concentration of C12:0 and C14:0. The fatty acid (FA) profile in breast muscle obtained from broilers fed diets with increasing insect fat inclusion showed a significant linear effect in terms of C12:0, C15:0, C18:2, C18:3n6, and total FAs. The proportion of dietary insect fat had a quadratic effect on meat color. The water-holding capacity indices have stayed consistent with the meat color changes. Throughout the experiment, favorable growth performance results were noticed in HI06. The present study confirmed that BSF larvae fat negatively affects the n3 level in meat. However, the physicochemical indices related to consumer acceptance were not altered to negatively limit their final decision, even when a relatively high inclusion of insect fat was used.

## 1. Introduction

The use of insects as an alternative protein or energy source in poultry nutrition has been frequently studied, mostly with satisfactory results [1,2]. Under experimental conditions, mainly full-fat or defatted insect meals are provided in animal diets, overlooking that the lipid content is the second most abundant nutrient in the invertebrate body, reaching up to 47% on a dry matter (DM) basis [3]. Dietary fat is obtained from invertebrate species, i.e., *Tenebrio molitor* (meal worm), *Zophobas morio* (super mealworm), and *Hermetia illucens* (black soldier fly; BSF), which may be considered substitutes for soybean and palm oils, which are environmentally harmful and commonly used in animal nutrition [4,5,6]. This is possible because the *H. illucens* larvae fat contains an energy density similar to that of soybean oil (9019 kcal apparent metabolizable energy corrected to zero nitrogen balance (AME_N_)/kg vs. 9000 AME_N_ kcal/kg) [7,8]. Thus, this feed material may replace soybean oil in broiler diets at a 1:1 ratio.

Based on the above, unsurprisingly, the use of BSF larvae fat alone or in combination with soybean oil does not impair the growth performance of broiler chickens [9], turkeys [4], or laying hens [10]. Additionally, nutrient digestibility coefficients are not decreased with dietary insect fat use because of the high lipase and amylase activity in the duodenum [4]. Furthermore, it is well documented that BSF larvae fat not only provides energy but may also be considered a functional feed material [1,11]. In particular, the high concentration of lauric acid as a predominant medium-chain fatty acid (MCFA) in BSF may positively affect the microbiota microecosystem of the birds’ gastrointestinal tract [4,12,13]. Furthermore, an indirect effect on immune system support can be observed [1].

However, to date, only a few articles have characterized BSF larvae fat as a factor that affects the final product quality in poultry. In general, the fatty acid (FA) profile of *H. illucens* is mostly characterized by saturated fatty acids (SFAs) (ca. 70%), such as lauric (C12:0), myristic (C14:0), palmitic (C16:0), and stearic acids (C18:0). Unsaturated fatty acids (UFAs), e.g., oleic acid (C18:1) and linoleic acid (C18:2), are in the minority [3]. However, it should be noted that both invertebrate lipid content and FA profile are highly dependent on the chemical composition of the substrate on which insects are reared and can be modified [14,15]. Nevertheless, lauric acid is synthesized by larvae de novo [16], and its concentration in the substrate does not affect its level in insect biomass [17]. The specification of *H. illucens* fat corresponds to the chemical quality of animal products, including rabbits [18] and various fish species [19,20]. Schiavone et al. [9] emphasized that the use of BSF larvae fat as a soybean oil replacement elevated the SFAs and reduced UFAs in broiler chicken breast meat. However, no effect on the *pectoralis major* muscle was observed (Day 0 and Day 9). Moreover, drip loss was not affected. However, Sypniewski et al. [9] did not find any adverse changes in triglyceride and cholesterol levels in the thigh and breast muscles of turkeys. No effects on the selected physicochemical quality of the leg and breast meat were noted when BSF larvae fat was used as a soybean oil substitute in broiler diets [21]. The FA composition of these products was highly affected.

Additionally, it should be mentioned that in the available literature, on the one hand, there is information about using BSF larvae fat in diets, mostly at approximately 5% of the diet. On the other hand, it is rare to use BSF larvae fat alone because soybean oil is simultaneously included in the diet. Hence, it is the effect of the dietary fat mixture that is assessed. Therefore, the current study aimed to evaluate the dose-dependent effect of BSF larvae fat inclusion in broiler chicken diets on breast meat quality. Furthermore, the presented study was conducted to emphasize critical challenges linked with the future application of insect-derived dietary fat in various amounts to poultry diets which may affect the final product quality, i.e., breast meat.

## 2. Materials and Methods

### 2.1. Ethics Statement

According to Polish law and the EU directive (no. 2010/63/EU), the experiments conducted within this study did not require the approval of the Local Ethics Committee for Experiments on Animals of Poznań. All of the procedures complied with the guidance on animal experimentation and the care of animals under study, and all efforts were made to minimize suffering.

### 2.2. Birds and Housing

The current study is a continuation of a previously published experiment conducted on broiler chickens that aimed to assess *H. illucens* larvae fat apparent metabolizable energy [7]. Four hundred 1-day-old male birds (Ross 308; average initial weight 37 g ± 0.5 g) obtained from a commercial hatchery were randomly allotted to 4 dietary treatments. In each group, 10 replicate pens with 10 birds per pen were used. Broilers were kept in floor pens (1 m × 1 m), and chopped wheat straw (7–15 cm) was used as litter. The pens were placed according to a completely randomized design in the center of a chicken house (no. 0161, Olszowa, Poland). To imitate intensive rearing conditions, the experimental pens were surrounded by 9000 unsexed chicks of the same origin. The experiment lasted 35 days. The environmental conditions were established as follows: at the beginning of the experiment, the temperature was set to 32–33 °C and then reduced by 2–3 °C each week. On the 28th day, the temperature was set to 21 °C and reduced to approximately 18 °C at the end of the test. The chicken house was equipped with artificial programmable fluorescent light, automatic electric heating and forced ventilation, which were arranged according to the recommendations of the Management Handbook [22] and the recommendations of the Council Directive (2007/43/EC). The birds were vaccinated in the hatchery against Infectious Bronchitis (Nobilis IB Ma5, and Nobilis IB 4/91, Intervet International B.V., Boxmeer, Netherland). During days 12 and 20 after hatching, chicks were vaccinated against Gumboro disease (AviPro PRECISE, Lohmann Animal GmbH, Cuxhave, Germany).

### 2.3. Broiler Diets

The composition and nutritive value of the experimental diets are shown in Table 1. Broiler chickens had *ad libitum* access to feed (one feed hopper per pen) and water (two nipple drinkers per pen). The maize–soy basal diet was formulated using NRC guidelines (1994) and administered from the bird age of 1 to 35 days. The diets were enriched with 30, 60, and 90 g/kg *H. illucens* larvae fat and provided in mash form, with all raw materials grounded using a disc mill (Skiold A/S, Denmark) at a 2.5 mm disc distance and mixed without heat treatment. The feed was produced in the Piast Pasze feed mill (Lewkowiec, Poland) according to ISO 9001:2008 procedures, and the preparation process occurred on a laboratory scale line equipped with a horizontal double band mixer (Zuptor, Gostyń, Poland) with roller mills (Skiold, Sæby, Denmark). No viscous cereals were used in the experimental diet to avoid reducing energy availability. Furthermore, no feed additives, such as exogenous enzymes, coccidiostats, and emulsifiers, were used. Insect fat production was fully described in Kierończyk et al. [4]. In short, *H. illucens* larvae were fed using fresh plant-origin feed materials, such as wheat middling, vegetable, and fruit mixture, according to EU Regulation 2009/1069, i.e., without animal-origin products. Larvae at the prepupal stage were harvested, sieved, washed, and air-dried (50 °C for 24 h). Furthermore, the *H. illucens* fat was extracted mechanically using the cold-press method (AP 14/22 oil press, Reinartz, Neuss, Germany) under the following conditions: 85 °C and 95% humidity. No refining process was used. Due to the solid state of the obtained fat before the experimental diet preparation, the fat was heated to 40 °C in a water bath W4415 (Laboplay, Bytom, Poland) until all fat was melted. The fatty acid composition of the *H. illucens* fat is shown in Table 2.

The design of the trial was set up as follows: HI0, basal diet without dietary fat inclusion; HI03, basal diet enriched with 30 g/kg *H. illucens* larvae fat; HI06, basal diet enriched with 60 g/kg *H. illucens* larvae fat; and HI09, basal diet enriched with 90 g/kg *H. illucens* larvae fat.

### 2.4. Data and Sample Collection

The birds and feed were weighed on Days 14, 21, 28, and 35. The body weight gain (BWG), feed intake (FI), and feed conversion ratio (FCR) were calculated for the following periods, i.e., 1–14 days, 15–21 days, 22–28 days, 29–35 days, and 1–35 days. For the growth performance results, the pen was defined as an experimental unit (*n* = 10). At the end of the trial, 10 randomly selected birds were slaughtered, followed by exsanguination (external jugular vein cut) after electrical stunning (STZ 6, PPHU KOMA, Poland). After bleeding, birds were placed in a scalding tank (approximately 2 min, 60 °C; Technologies 4All Group, Kępno, Poland) and de-feathered using an automatic turning machine (Technologies 4All Group, Kępno, Poland). Next, the right breast muscle (*pectoralis major*) from 10 randomly selected birds (*n* = 10) was separated and immediately frozen on dry ice and stored at −20 °C for further chemical analyses performed at the Department of Chemistry, Poznań University of Life Sciences (Poznań, Poland).

### 2.5. Physico-Chemical Analysis

The left breast (*n* = 10) was used for the retail display storage test. The pH measurement was performed *postmortem* (after 20 min, 1 h, and 24 h) using a ScienceLine Micro pH combination electrode N 6000 BNC (Schott SI Analytics, Mainz, Germany) inserted into the cranial end of the intact fillet and an 1100 H pH meter (VWR International, Leuven, Belgium). Each measurement was performed in duplicate, and then the mean was utilized. A colorimeter (MiniSan EZ, HunterLab, Murnau, Germany) was used for color measurement according to the CIELab system [23], including L*, lightness; a*, redness index (+/− red–green); and b*, yellowness index (+/− yellow–blue). Meat color analysis was performed at room temperature (20 °C) with the device resting on samples in the horizontal plane. Additionally, based on the abovementioned coordinates, the chroma saturation index and hue angle were calculated according to the following equation, i.e., chroma (C*) = (a*^2^ + b*^2^)^1/2^ and hue angle (H°) = tan^−1^ (b*/a*) [23] and expressed in degrees (°). The color was measured three times on the internal side of each raw pectoral muscle, and the mean from triplicate determination was used as an experimental unit. For drip loss determination, a sample weighing approximately 40 g (*n* = 10), obtained from the caudal portion of each fillet, was suspended in a plastic box at 4 °C. After 24 h and 7 days, the samples were cleaned to eliminate the extra surface fluids and weighed, and the drip loss was calculated as the percentage of weight lost by the sample during the refrigerated storage interval [24]. Salt-induced water uptake was measured 24 and 48 h postmortem, according to Bowker et al. [25]. Briefly, 10 g of the chopped muscle samples were mixed with 15 mL of cold 0.6 M NaCl solution in 50 mL centrifuge tubes using a vortex mixer for 1 min, stored at 4 °C for 15 min, and centrifuged at 7000× *g* for 15 min at 4 °C. The excess liquid was decanted, and the sample was finally weighed. Salt-induced water uptake was calculated as the percentage of weight gained by the sample. The composition of the fatty acid (FA) profile of the *H. illucens* larvae fat and experimental diets (Table 2) was determined in duplicate using a gas chromatograph–mass spectrometer (Varian 450-GC, The Netherlands) coupled to a mass spectrometer (Varian 320-MS, Walnut Creek, CA, USA) according to the method described by Stuper-Szablewska et al. [26]. Additionally, FA determination was performed in other selected dietary fats, such as soybean oil, rapeseed oil, palm oil, palm kernel fatty acid distillers, poultry fat, pig lard, beef tallow, *T. molitor* larvae oil, and *Z. morio* larvae fat, to evaluate the variation among them. All of the abovementioned dietary fats were purchased from Piast Pasze feed mill and HiProMine S.A. as commonly used energy sources in animal nutrition or examples of insect dietary fat examined in other studies [3,6]. The atherogenic index (AI) and thrombogenic index (TI) were computed according to Gao et al. [27] based on the following equations:

$$Atherogenic\;index=\left(\frac{C12:0+4\;x\;C14:0+C16:0}{\sum MUFA+\sum \left(n-6\right)+\sum \left(n-3\right)}\right)$$

$$Thrombogenic\;index=\left(\frac{C14:0+C16:0+C18:0}{0.5\;x\;\sum MUFA+0.5\;x\;\sum \left(n-6\right)+3\;x\;\sum \left(n-3\right)+\frac{\sum \left(n-3\right)}{\sum \left(n-6\right)}}\right)$$

### 2.6. Statistical Analyses

The experiment had a completely randomized design. In terms of growth performance, the pen was defined as an experimental unit, while the individual bird (one randomly chosen broiler per pen) was used for the selected physicochemical traits of meat.

The statistical analyses were performed using RStudio (v. 2022.12.0 + 353; 2022 RStudio, Inc., Boston, MA, USA). All of the data were tested for normal distributions using the Shapiro–Wilk test. Next, the collected data were tested by one-way ANOVA. The results determined for the treatments were compared using the Bonferroni test. Orthogonal polynomial contrasts (linear and quadratic effects) were used to determine the effect of increasing *H. illucens* larvae fat inclusion in the broiler chicken diets. Differences were considered significant at *p* < 0.05. All data are presented as the means with standard error of the mean (SEM). Principal component analysis (PCA) was computed using the following packages, *stats* (3.6.2) and *ggbiplot* (0.55), to show the relationship between the fatty acid composition and selected dietary fats.

## 3. Results

### 3.1. Hermetia illucens Fatty Acid Profile

**Figure 1:**
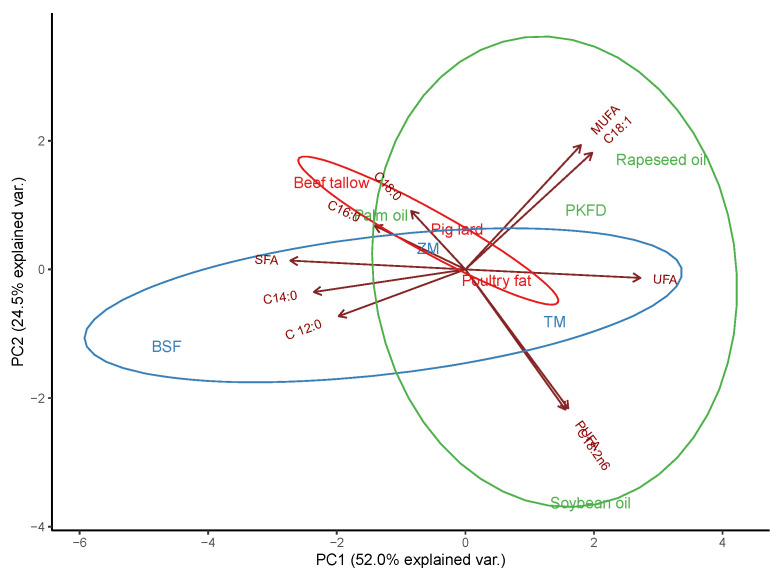
Principal component analysis (PCA) of fatty acid allocation in the selected dietary fat of animal (red), plant (green), and insect (blue) origin. BSF = black soldier fly (*Hermetia illucens*), TM = *Tenebrio molitor* (mealworm), ZM = *Zophobas morio* (super mealworm), PKFD = palm kernel fatty distillers, SFA = saturated fatty acids, UFA = unsaturated fatty acids, MUFA = monounsaturated fatty acids, PUFA = polyunsaturated fatty acids. Based on the results obtained by Benzertiha et al. [3,6] and a project mentioned in the Founding section.

The PCA showed a clear differentiation between the plant-, animal-, and insect-origin dietary fats (Figure 1). Because the first two principal components explained 76.5% of the variance, PC1 and PC2 were plotted. In particular, BSF larvae fat has a strong relationship with SFA as the result of high concentrations of C12:0 and C14:0, thus differing from soybean and rapeseed oils, which are characterized by high PUFA (C18:2 n-6) and MUFA levels (C18:1), respectively. Other insect species, such as *T. molitor* and *Z. morio*, which are not rich sources of lauric acid, remain more comparable to other animal-origin dietary fats (pig lard and poultry fat) and palm oil linked with SFAs, essentially C16:0 and C18:0.

### 3.2. Selected Physico-Chemical Meat Traits

The increasing inclusion of *H. illucens* larvae fat in the broiler chicken diets highly affected the fatty acid profile of the breast meat (Table 3). A significant linear effect (*p* < 0.001) was observed in the case of lauric acid (C12:0), pentadecylic acid (C15:0), linoleic acid (C18:2), and gamma-linolenic acid (C18:3n6). The same response (*p* < 0.001) was noticed in summarized FAs, i.e., SFA, UFA, MUFA, PUFA, PUFA/SFA, and n6, n3, and their ratio. Other determined fatty acids were characterized by the quadratic effect (*p* < 0.001), where each SFA increased in a dose-dependent manner, except for C18:0 (*p* = 0.033) and C20:0 (*p* < 0.001), which significantly reduced their level. Additionally, the concentration of oleic acid (C18:1) and gondoic acid (C20:1) in the breast decreased according to increased insect fat levels in the diets. Furthermore, palmitoleic acid (C16:1), heptadecenoic acid (C17:1), and eicosadienoic acid (C20:2) were not detected in the HI0 group. The concentration of these FAs varied in terms of *H. illucens* larvae fat inclusion, i.e., decreased (C20:2), increased (C16:1), or the highest dietary fat level resulted in no significant difference between other treatments (C17:1). Furthermore, the summarized fatty acids presented as UFAs, PUFAs, PUFA/SFA, and n6 showed a significant and linear increase (*p* < 0.001) when birds were fed with *H. illucens* fat in higher amounts (60 and 90 g/kg). n6/n3 exhibited increased values in a dose-dependent manner. The opposite results were observed for SFAs and MUFAs, where a significant (*p* < 0.001) reduction was noted in the treatments with the highest levels of supplementation (HI06 and HI09). Additionally, decreasing n3 FA concentrations were observed according to diets enriched by *H. illucens* larvae fat in a dose-dependent manner. Moreover, the AI exhibited a quadratic trend, i.e., the addition of insect fat in amounts of 30, 60, and 90 g/kg of diet caused an increased value of this trait in contrast to the HI0 group. The TI was not affected (*p* = 0.128) by the experimental factor.

The color of breast muscle was quadratically affected (*p* <0.05) by insect fat inclusion in broiler chicken diets (Table 4). The highest (*p* = 0.010) lightness value of meat was observed in the HI03 and HI06 treatments, contrary to the group where no dietary fat was used. Simultaneously, meat from HI09 did not differ among groups in terms of lightness. The redness index was significantly reduced (*p* = 0.025) with the use of 90 g/kg of dietary *H. illucens* larvae fat, while the implementation of insect energy sources up to 60 g/kg of diet did not show any changes. No significant differences in the yellowness index or chroma were observed between treatments with dietary insect fat inclusion, contrary to HI0, which showed the lowest value (*p* = 0.024 and *p* = 0.027, respectively). Contrary to other color parameters, the hue angle was characterized by a linear response (*p* < 0.001). The lowest value was observed in the HI0 group, while the addition of *H. illucens* larvae fat to diets resulted in higher and equal values among the remaining groups. The increasing inclusion of insect fat did not result in any significant changes in terms of the pH value of chicken breast meat (*p* > 0.05). However, drip loss was affected linearly (24 h after slaughter; *p* = 0.010) or quadratically (7 days after slaughter; *p* = 0.035) and decreased in a dose-dependent manner. Salt-induced water uptake linearly increased (24 h; *p* = 0.002) or decreased (48 h; *p* = 0.004) with increasing dietary fat levels in the diet.

### 3.3. Growth Performance

The growth performance results showed a quadratic response, except for the FCR calculated for the 22–28 d period, which exhibited a linear effect (Table 5). Unsurprisingly, the lowest (*p* = 0.001) BWG in each period was observed in the treatment without dietary fat inclusion. The most positive effect on BWG was observed in the HI03 and HI06 groups. Only in the case of the 22–28 days period did HI09 not differ from the other treatments supplemented with *H. illucens* fat. In general, the FI was reduced (*p* < 0.001) by the 90 g/kg addition of insect fat. Only in the 22–28 days period was FI limited by the HI0 group (*p* = 0.001). Additionally, there were no observed changes in FI between these treatments (HI0 vs. HI09) in the 29–35 days period or in the entire experiment (1–35 days). Similarly, throughout the whole experiment, FI was at the same level (*p* < 0.001) when 30 or 60 g/kg insect fat was added to the birds’ diets. A favorable FCR value was noticed in each experimental period (*p* = 0.001) in the HI06 and HI09 treatments, except for the 29–35 days period, where the group with the highest addition of insect fat did not differ from HI0 and HI03. Moreover, the lack of dietary fat administration in broiler nutrition resulted in elevated (*p* = 0.001) FCR values in all periods, as well as the entire experiment.

## 4. Discussion

*Hermetia illucens* larvae fat and the effects of the nutritional value of the substrate provided to insects during rearing on its quality are well documented in the available literature [3,14]. In general, the results of the present study confirmed the previously reported FA profile of BSF larvae fat [28], where the predominant SFAs are lauric acid (C12:0) and myristic acid (C14:0). In addition, the concentration of palmitic acid (C16:0) in the BSF larvae fat was also high; however, it is distinctive for each animal-origin fat and palm oil as an exception [29]. Furthermore, it should be highlighted that due to the specific lipid characterization, there were clear differences observed between various insect dietary fats in the promotion of SFAs (BSF larvae), PUFAs (*T. molitor* larvae), and MUFAs (*Z. morio*). Nevertheless, the above and elevated levels of oleic acid (C18:1) and linoleic acid (C18:2) in insect biomass resulted in a spatial arrangement between vegetable oils and animal fats in terms of FA composition. This is supported by the results of Sosa and Fogliano [30]. Contrary to other authors [9,31,32], in the present study, the concentration of lauric acid in BSF larvae fat was relatively low. Usually, its level ranges between 21% and approximately 60% of total FAs [31,32,33]. Hoc et al. [16] reported that the level of C12:0 in BSF biomass is related to C18-FA accumulation in the insect-rearing substrate, as well as carbohydrate availability (glycolysis as a donor of pyruvate and its conversion into acetyl-CoA). In the present study, larvae were fed vegetable and fruit wastes with wheat middling according to Kierończyk et al. [14]. This study confirms that high wheat inclusion decreases the lauric acid concentration in larval biomass and should be avoided in BSF nutrition. This is particularly true when its functional effect is preferred [34]. Additionally, the increased carbohydrate [35] and protein inclusion [36], their ratio [37], and reduced fiber content [32,38] in rearing substrate may cause crude fat, as well as lauric acid enhancement in BSF larvae biomass.

Unsurprisingly, in the present study, the FA profile of broilers’ breast meat mirrored the chemical composition of the experimental diets. In the available literature, the same effect was observed in poultry, fish, and rabbit products [18,39,40]. However, the current experiment indicated that the increasing *H. illucens* larvae fat in bird diets exhibits mainly a linear response in terms of summarized FAs and a quadratic response in the case of specific SFAs, as well as UFAs, with some exceptions that showed a linear effect, e.g., C12:0, C15:0, and C18-PUFAs. Interestingly, the considerable amount (60 and 90 g/kg) of BSF larvae fat in broilers’ diets resulted in a positive increase in PUFA concentration in the breast meat, particularly as an effect of enhanced linolenic acid (C18:2). In contrast, MUFA levels were reduced by oleic acid (C18:1). Eventually, the elevated UFA level and reduction in SFAs suggest a beneficial effect for consumers. Nevertheless, the increased concentration of linolenic acid should not be related to BSF larvae fat, which was decreased in birds’ diets in a dose-dependent manner. It should be emphasized that FAs in tissues may be synthesized de novo [41] because feed intake did not increase in birds fed higher BSF fat, as Tor et al. [42] suggested. Additionally, it is well documented that increased PUFAs inhibit MUFA synthesis in the liver by lowering 9-desaturase activity [43]. Nevertheless, the PUFA to SFA ratio was established at an acceptable level (ca. ≤2). According to Turley and Thompson [44], tumor formation may be promoted by a high PUFA/SFA ratio, i.e., ≥3, or an atherogenic effect by low values (≤0.33). This is supported by the atherogenic and thrombogenic indexes, which were at a similar level to those obtained from the meat tissue belonging to birds fed diets with plant-origin oils [5,6]. Additionally, the n6/n3 ratio, as an index that may be linked with various physiological disorders, such as atherosclerosis and coronary heart disease, showed values close to those presented by Milićević et al. [43] or Kierończyk et al. [5]. However, it should be highlighted that generally, low availability of n3 polyunsaturated fatty acids in BSF larvae fat and further in the experimental diets resulting in meat n3 deficiency can be considered a crucial drawback. The deficit mentioned above in the BSF larvae biomass may be linked with wheat middlings used for dry matter regulation in the rearing substrate [14]. Thus, further research must be undertaken in terms of enhancing the n3 PUFAs in BSF larvae biomass and directly improving the quality of the final product.

To date, the effect of invertebrate biomass on the quality of poultry products has primarily been evaluated using both full-fat and defatted insect meals. It should be emphasized that in those experimental designs, the insect fat addition ranges from approximately 0.5% to 2.5% in the bird diets [45,46,47,48,49,50]. Thus, its impact on birds’ meat is limited and only partially linked to insect fat because soybean oil is often used in parallel in experimental diets to optimize energy density. The present study confirmed previous reports [9,21] that BSF larvae fat does not negatively affect the pH of the meat of broilers even when added in relatively high amounts (90 g/kg). The regular ultimate pH value of meat (determined 24 h after slaughter) varied between 5.7 and 6.1, indicating proper technological quality [51]. Below this range, meat is defined as acidic, and above this range, it is defined as dark, firm, and dry (DFD). It should be noted that no influence of increased dietary fat was noted on pH measured at 20 min and 60 min. Interestingly, a relatively high brightness (L*) was observed with increased dietary fat when the pH value was also high, i.e., >6.0. Mir et al. [52] highlighted the opposite trend of decreasing lightness of meat caused by minimal protein denaturation. Nevertheless, it is well known that the implementation of animal-origin dietary fat in poultry nutrition results in enhanced light color and yellowness (b*) of breast meat, in contrast to vegetable oils [53]. Additionally, the elevated brightness of meat may result from moisture content, as examined by two separate methods, i.e., drip loss and salt-induced water uptake. First, the exudation of water was evaluated, and the second technique was used to measure water absorption. In the present study, the drip loss measurement exhibited lower values from muscle (at 24 h and 7 days after slaughter) and higher salt-induced water uptake (HI09, 48 h). This result is in line with the high correlation between L* and the water-holding capacity indices (|r| = 0.71–0.83) [25]. Furthermore, the yellowness (b*), hue angle (H°), and chroma (C*) did not differ between birds fed different levels of BSF larvae fat. However, the significantly higher results between these treatments and the group without dietary fat inclusion may be a result of fat deposition in the muscle. In general, the obtained physicochemical indices results should not affect final consumer motivation; however, it is important to continue experiments on the effect of insect dietary fat on the quality of final poultry products.

In the present study, the growth performance results cannot be compared between treatments due to the varying energy densities in the experimental diets. Primarily, the increased inclusion of dietary fat was established to evaluate the regression models for metabolizable energy value calculation of BSF larvae fat, which are available and presented in Kierończyk et al. [7]. Nevertheless, the growth performance results confirm that the BSF larvae fat energy level is comparable to that of soybean oil used in broiler chicken diets. The final FCR results in HI06 and HI 09 agree with the performance objectives of Aviagen Ross 308 (2022). However, due to the significant decrease in BWG in the 9% supplemented group, i.e., 70 g (HI06 vs. HI09), the preferred level of insect fat is 6%.

## 5. Conclusions

The present study confirmed that *H. illucens* larvae fat highly affected the fatty acid profile of the broilers’ breast muscle, where mainly the crucial n3 level drawback was noticed. Therefore, it is recommended to continue research to enrich insect biomass in fatty acids related to improving human health status. However, the physicochemical indices related to consumer willingness were not altered to limit their final decision, even when a high amount of *H. illucens* larvae fat was used. Finally, the growth performance results suggested the possible implementation of a relatively high amount (60 g/kg) of insect fat in broiler chickens’ diets to meet hybrid producer performance objectives.

## Figures and Tables

**Table 1:** Composition of the broiler chicken experimental diets (g kg^−1^, as-fed basis).

Ingredients, g kg^−1^	^1^ HI0	^2^ HI03	^3^ HI06	^4^ HI09
Maize	657	657	657	657
Soybean meal, 468 g CP/kg	306	306	306	305
Monocalcium phosphate	11.9	11.9	11.9	11.9
Limestone	10.5	10.5	10.5	10.5
*Hermetia illucens* larvae fat	0.00	30.0	60.0	90.0
NaCl	2.60	2.60	2.60	2.60
Na_2_SO_4_	1.20	1.20	1.20	1.20
^5^ Mineral-vitamin premix	3.00	3.00	3.00	3.00
L-Lysine	1.80	1.80	1.80	1.80
L-Methionine	2.30	2.30	2.30	2.30
L-Threonine	0.60	0.60	0.60	0.60
L-Valine	0.10	0.10	0.10	0.10
TiO_2_	3.00	3.00	3.00	3.00
Analyzed nutritive value, g kg^−1^				
Dry matter	884	888	893	896
Gross energy, MJ/kg	16.4	17.0	18.0	18.5
Crude protein	208	222	209	207
Crude fat	2.65	4.31	8.2	10.2

^1^ HI0, basal diet without dietary fat inclusion; ^2^ HI03, basal diet enriched with 30 g/kg *H. illucens* larvae fat; ^3^ HI06, basal diet enriched with 60 g/kg *H. illucens* larvae fat; ^4^ HI09, basal diet enriched with 90 g/kg *H. illucens* larvae fat, ^5^ Provided the following per kilogram of diet: vitamin A, 11,000 IU; cholecalciferol, 2500 IU; vitamin E, 50 mg; menadione, 2.50 mg; vitamin B_12_, 0.02 mg; folic acid, 1.0 mg; choline, 300 mg; D-pantothenic acid, 13.6 mg; riboflavin, 7.0 mg; niacin, 41.7 mg; thiamine, 2.0 mg; D-biotin, 0.20 mg; pyridoxine, 4.0 mg; ethoxyquin, 0.1 mg; Mn (MnO_2_), 60 mg; Zn (ZnO), 95 mg; Fe (FeSO_4_), 45 mg; Cu (CuSO_4_), 20 mg; I (CaI_2_O_6_), 0.6 mg; and Se (Na_2_SeO_3_), 0.35 mg.

**Table 2:** Fatty acid profile of *Hermetia illucens* fat and experimental diets (g/100 g fatty acids) fed to broiler chickens.

Item	^1^ HI	^2^ HI0	^3^ HI03	^4^ HI06	^5^ HI09
Saturated					
C8:0	0.12	0.00	0.02	0.05	0.1
C10:0	0.04	0.00	0.01	0.02	0.02
C12:0	20.3	0.00	3.86	6.1	8.4
C14:0	6.74	0.00	1.6	2.2	3.0
C16:0	26.09	11.5	13.2	15.5	16.9
C18:0	2.46	2.80	2.60	2.70	2.68
C21:0	0.00	0.00	0.1	0.1	0.00
C24:0	0.00	0.00	0.1	0.1	0.00
Monosaturated					
C16:1n7	24.1	0.00	1.48	1.9	2.3
C18:1 n9	3.45	34.8	32.9	29.9	26.4
Polysaturated					
C18:2n6 (linoleic acid)	16.7	50.8	44.0	41.33	40.1
C18:3n3 (linolenic acid)	0.00	0.10	0.10	0.10	0.10
Total	100	100	100	100	100
^6^ SFA	55.75	14.3	21.52	26.77	31.1
^7^ UFA	44.25	85.7	78.48	73.23	68.9
^8^ MUFA	27.55	34.8	34.38	31.8	28.7
^9^ PUFA	16.70	50.9	44.1	41.43	40.2
PUFA/SFA	0.30	3.56	2.05	1.55	1.29

^1^ HI, *Hermetia illucens* larvae fat; ^2^ HI0, basal diet without dietary fat inclusion; ^3^ HI03, basal diet enriched with 30 g/kg *H. illucens* larvae fat; ^4^ HI06, basal diet enriched with 60 g/kg *H. illucens* larvae fat; ^5^ HI09, basal diet enriched with 90 g/kg *H. illucens* larvae fat; ^6^ SFA, saturated fatty acids; ^7^ UFA, unsaturated fatty acids; ^8^ MUFA, monounsaturated fatty acids; ^9^ PUFA, polyunsaturated fatty acids.

**Table 3:** Effect of increasing dose inclusion of *Hermetia illucens* larvae fat in the broiler chicken diets on the breast meat fatty acid profile (35 days).

Item	Treatment				^5^ SEM	*p*	Linear Effect	Quadratic Effect
	^1^ HI0	^2^ HI03	^3^ HI06	^4^ HI09				
*Saturated*								
C12:0	^6^ ND ^c^	0.14 ^b^	0.21 ^b^	0.30 ^a^	0.02	<0.001	<0.001	0.378
C14:0	0.54 ^d^	0.86 ^c^	0.96 ^b^	1.08 ^a^	0.03	<0.001	<0.001	<0.001
C15:0	ND ^d^	0.06 ^c^	0.13 ^b^	0.24 ^a^	0.02	<0.001	<0.001	0.069
C16:0	15.3 ^d^	14.6 ^c^	14.2 ^b^	13.9 ^a^	0.09	<0.001	<0.001	<0.001
C17:0	0.28 ^c^	0.19 ^b^	0.11 ^a^	0.09 ^a^	0.01	<0.001	<0.001	<0.001
C18:0	4.73 ^ab^	4.88 ^a^	4.71 ^ab^	4.66 ^b^	0.03	0.026	0.082	0.033
C20:0	0.15 ^c^	0.21 ^a^	0.18 ^ab^	0.16 ^bc^	0.01	<0.001	0.751	<0.001
*Unsaturated*								
C16:1	ND ^c^	1.24 ^b^	1.29 ^b^	1.35 ^a^	0.09	<0.001	<0.001	<0.001
C17:1	ND ^c^	1.55 ^a^	1.42 ^b^	1.50 ^ab^	0.11	<0.001	<0.001	<0.001
C18:1	39.3 ^a^	37.0 ^b^	36.1 ^c^	35.7 ^c^	0.24	<0.001	<0.001	<0.001
C18:2	36.0 ^b^	36.4 ^b^	38.1 ^a^	38.6 ^a^	0.20	<0.001	<0.001	0.887
C18:3n6	0.18 ^b^	0.29 ^a^	0.30 ^a^	0.39 ^a^	0.02	<0.001	<0.001	0.728
C18:3n3	2.75 ^a^	2.39 ^b^	2.17 ^c^	1.95 ^d^	0.05	<0.001	<0.001	0.178
C20:1	0.78 ^a^	0.09 ^b^	0.07 ^bc^	0.05 ^c^	0.05	<0.001	<0.001	<0.001
C20:2	ND ^c^	0.12 ^a^	0.09 ^b^	0.08 ^b^	0.01	<0.001	<0.001	<0.001
*Summarized*								
^7^ SFA	21.0 ^a^	20.9 ^a^	20.5 ^b^	20.4 ^b^	0.06	<0.001	<0.001	0.980
^8^ UFA	79.0 ^b^	79.1 ^b^	79.5 ^a^	79.6 ^a^	0.06	<0.001	<0.001	0.980
^9^ MUFA	40.1 ^a^	39.8 ^a^	38.9 ^b^	38.6 ^b^	0.14	<0.001	<0.001	0.925
^10^ PUFA	38.9 ^b^	39.1 ^b^	40.6 ^a^	40.9 ^a^	0.17	<0.001	<0.001	0.689
PUFA/SFA	1.85 ^b^	1.87 ^b^	1.98 ^a^	2.01 ^a^	0.01	<0.001	<0.001	0.857
n6	36.2 ^b^	36.8 ^b^	38.5 ^a^	39.1 ^a^	0.21	<0.001	<0.001	0.824
n3	2.75 ^a^	2.69 ^b^	2.17 ^c^	1.95 ^d^	0.05	<0.001	<0.001	0.178
n6/n3	13.2 ^d^	15.4 ^c^	18.1 ^b^	20.1 ^a^	0.46	<0.001	<0.001	0.948
Atherogenic index	0.221 ^b^	0.230 ^a^	0.229 ^a^	0.232 ^a^	<0.01	<0.001	<0.001	0.037
Thrombogenic index	0.443	0.446	0.439	0.438	<0.01	0.128	0.058	0.472

^1^ HI0, basal diet without dietary fat inclusion; ^2^ HI03, basal diet enriched with 30 g/kg *H. illucens* larvae fat; ^3^ HI06, basal diet enriched with 60 g/kg *H. illucens* larvae fat; ^4^ HI09, basal diet enriched with 90 g/kg *H. illucens* larvae fat; ^5^ SEM, standard error of the mean; ^6^ ND, not detected, ^7^ SFA, saturated fatty acids; ^8^ UFA, unsaturated fatty acids; ^9^ MUFA, monounsaturated fatty acids; ^10^ PUFA, polyunsaturated fatty acids. ^a–d^ Means within a row not sharing a common superscript differ at *p* < 0.05. Means represent 10 randomly chosen birds (*n* = 10).

**Table 4:** Effect of increasing dose inclusion of *Hermetia illucens* larvae fat in broiler chicken diets on the selected physicochemical quality traits of breast meat (35 days).

Item	Treatment				^5^ SEM	*p*	Linear Effect	Quadratic Effect
	^1^ HI0	^2^ HI03	^3^ HI06	^4^ HI09				
Color								
^6^ L*	64.1 ^b^	70.4 ^a^	71.3 ^a^	69.8 ^ab^	0.83	0.002	0.009	0.010
^7^ a*	5.84 ^a^	6.30 ^a^	5.86 ^a^	2.54 ^b^	0.46	0.002	0.007	0.025
^8^ b*	13.3 ^b^	20.3 ^a^	20.6 ^a^	21.6 ^a^	0.81	<0.001	<0.001	0.024
Chroma (C*)	14.6 ^b^	21.4 ^a^	21.5 ^a^	22.0 ^a^	0.82	<0.001	0.001	0.027
Hue angle (H°)	67.9 ^b^	72.8 ^a^	74.1 ^a^	82.8 ^a^	1.23	<0.001	<0.001	0.305
pH								
20 min	6.41	6.33	6.36	6.47	0.29	0.198	0.472	0.100
60 min	5.86	5.98	6.00	5.97	0.03	0.131	0.124	0.185
24 h	5.81	5.77	5.75	5.76	0.02	0.407	0.295	0.402
Drip loss, %								
24 h	3.31 ^a^	2.80 ^b^	3.00 ^ab^	2.73 ^b^	0.07	0.023	0.010	0.343
7 days	5.97 ^a^	4.71 ^b^	4.86 ^b^	4.55 ^b^	0.13	<0.001	<0.001	0.035
^9^ Salt, %								
24 h	15.3 ^a^	12.9 ^ab^	10.6 ^ab^	8.97 ^b^	0.77	0.006	0.002	0.788
48 h	6.39 ^b^	6.93 ^ab^	7.88 ^ab^	9.64 ^a^	0.43	0.012	0.004	0.438

^1^ HI0, basal diet without dietary fat inclusion; ^2^ HI03, basal diet enriched with 30 g/kg *H. illucens* larvae fat; ^3^ HI06, basal diet enriched with 60 g/kg *H. illucens* larvae fat; ^4^ HI09, basal diet enriched with 90 g/kg *H. illucens* larvae fat; ^5^ SEM, standard error of the mean; ^6^ L*, lightness; ^7^ a*, redness index; ^8^ b*, yellowness index; ^9^ Salt, salt-induced water uptake. ^a,b^ Means within a row not sharing a common superscript differ at *p* < 0.05. Means represent 10 randomly chosen birds (*n* = 10).

**Table 5:** The effect of increasing inclusion of *Hermetia illucens* larvae fat in broiler chicken diets on their growth performance.

Item	Treatment				^5^ SEM	*p*	Linear Effect	Quadratic Effect
	^1^ HI0	^2^ HI03	^3^ HI06	^4^ HI09				
^6^ BWG, g								
0–14 days	325 ^c^	352 ^ab^	356 ^a^	340 ^b^	2.32	<0.001	0.005	<0.001
15–21 days	344 ^c^	380 ^ab^	393 ^a^	369 ^b^	2.89	<0.001	<0.001	<0.001
22–28 days	432 ^b^	470 ^a^	486 ^a^	486 ^a^	3.69	<0.001	<0.001	<0.001
29–35 days	638 ^c^	689 ^ab^	702 ^a^	673 ^b^	3.95	<0.001	<0.001	<0.001
1–35 days	1739 ^c^	1891 ^ab^	1938 ^a^	1868 ^b^	10.23	<0.001	<0.001	<0.001
^7^ FI, g								
0–14 days	447 ^a^	446 ^a^	439 ^a^	417 ^b^	2.07	<0.001	<0.001	<0.001
15–21 days	520 ^a^	532 ^a^	528 ^a^	492 ^b^	2.91	<0.001	<0.001	<0.001
22–28 days	633 ^b^	662 ^a^	667 ^a^	654 ^a^	2.82	<0.001	0.003	<0.001
29–35 days	1057 ^bc^	1089 ^a^	1080 ^ab^	1051 ^c^	4.07	<0.001	0.421	<0.001
1–35 days	2656 ^bc^	2729 ^a^	2714 ^ab^	2613 ^c^	9.62	<0.001	0.054	<0.001
^8^ FCR, g:g								
0–14 days	1.38 ^a^	1.27 ^b^	1.23 ^c^	1.23 ^c^	0.01	<0.001	<0.001	<0.001
15–21 days	1.51 ^a^	1.40 ^b^	1.34 ^c^	1.33 ^c^	0.01	<0.001	<0.001	<0.001
22–28 days	1.47 ^a^	1.41 ^b^	1.37 ^bc^	1.35 ^c^	0.01	<0.001	<0.001	0.194
29–35 days	1.66 ^a^	1.58 ^b^	1.54 ^c^	1.56 ^ab^	0.01	<0.001	<0.001	<0.001
1–35 days	1.53 ^a^	1.44 ^b^	1.40 ^c^	1.40 ^c^	0.01	<0.001	<0.001	<0.001

^1^ HI0, basal diet without dietary fat inclusion; ^2^ HI03, basal diet enriched with 30 g/kg *H. illucens* larvae fat; ^3^ HI06, basal diet enriched with 60 g/kg *H. illucens* larvae fat; ^4^ HI09, basal diet enriched with 90 g/kg *H. illucens* larvae fat; ^5^ SEM, standard error of the mean; ^6^ BWG, body weight gain; ^7^ FI, feed intake; and ^8^ FCR, feed conversion ratio. ^a–c^ Means within a row not sharing a common superscript differ at *p* < 0.05. Means represent 10 pens of 10 birds each (*n* = 10).

## Data Availability

All raw data obtained in this study are deposited in an official repository (https://doi.org/10.18150/KBXUWB (accessed on 15 February 2023)).
